# ﻿*Pternopetalumshunhuangensis* (Apiaceae), a new species from Hunan, China

**DOI:** 10.3897/phytokeys.253.142516

**Published:** 2025-03-12

**Authors:** Wei Zhou, Zi-Lin Feng, Long-Ping Tang, Du Deng, Bao-cheng Wu, Lei Wu

**Affiliations:** 1 Jiangsu Key Laboratory for the Research and Utilization of Plant Resources, Institute of Botany, Jiangsu Province and Chinese Academy of Sciences (Nanjing Botanical Garden Mem. Sun Yat-Sen), Nanjing 210014, Jiangsu, China Institute of Botany, Jiangsu Province and Chinese Academy of Sciences (Nanjing Botanical Garden Mem. Sun Yat-Sen) Nanjing China; 2 College of Forestry, Central South University of Forestry and Technology, Changsha 410004, China Central South University of Forestry and Technology Changsha China; 3 Hunan Dong’an Shunhuang Mountain National Nature Reserve, Yongzhou 425901, China Hunan Dong'an Shunhuang Mountain National Nature Reserve Yongzhou China; 4 College of Life and Environmental Sciences, Hunan University of Arts and Science, Changde 415000, China Hunan University of Arts and Science Changde China

**Keywords:** Apiaceae, China, new species, *Pternopetalum*

## Abstract

Based on field investigations, morphological and molecular systematic studies, a new species, *Pternopetalumshunhuangensis* (Apiaceae) from Hunan Province, China is described. Diagnostic morphological characters, full description and a detailed illustration are provided. The differences between *P.shunhuangensis* and morphologically similar species *P.tanakae* are presented and discussed. Since no population assessment of this species in its whole distribution area is made, it is best to assign a conservation status of ‘Data Deficient’ (DD) for this species.

## ﻿Introduction

*Pternopetalum* Franch. (Apiaceae), including ca. 25 species, is endemic to East Asia (Shan and Pu 1978; Pu 1985; Wu et al. 2006). Typical characteristics of this genus are petals saccate at base, umbellules with 2–5 flowers, and rays reflexed in fruit. In Wang’s revision (Wang 2007), the shape of the underground part, leaf morphology, the position of umbels, and the characteristics of flowers and fruits are important traits for delimiting *Pternopetalum* species.

With 23 species, 21 of which are endemic (Pu and Phillippe 2005), China is undoubtedly the center of diversity and endemism for *Pternopetalum*. Wang (2012) recognized only 15 species in the genus, while Pimenov (2017) considered it the eighth largest genus of Apiaceae in China, comprising 21 species. After Wang’s revision, three new species and one new combination of *Pternopetalum* were described from China (Tan et al. 2014, 2015; Zhong et al. 2018; Ye et al. 2020). Even the species *P.arunachalense* Bhaumik & P. Satyanar. which was published by Indian scholars Bhaumik and Satyanarayana (2014) was found in Southern Xizang according to the officially claimed boundary.

Species of *Pternopetalum* are mainly distributed in the southwest area of China, especially in Sichuan and Yunnan (Su and Sheh 2001). However, Hunan Province in Central China is also one of the concentrated distribution areas of this genus. There are ten species of *Pternopetalum* in Hunan, making it the largest genus of Apiaceae in the province (Sun et al. 2010).

During an arduous journey to the Hunan Dong’an Shunhuang Mountain National Nature Reserve in April 2024, we found an interesting population of *Pternopetalum* with flowers and young fruits. The species possesses unusual cauline leaves that are homomorphic with the basal leaves, sometimes even larger. In July and October, we returned to the locality and collected specimens with mature fruits. According to the characters of fruit, the species obviously belongs to P.sect.Pteridophyllae Wolff due to its minute calyx teeth and filiform ribs, but the homomorphic basal and cauline leaves differentiate it from all the other species of the section. Its relatively small habit and distinct fruit characteristics, also distinguish it from all the species in the other section, i.e., sect. Denterioideae Wolff.

After thorough consultation of the relevant literature and herbarium investigations, and comparison with morphologically similar species, we are confident that this is a new species of *Pternopetalum*.

## ﻿Materials and methods

### ﻿Morphological observation

Specimens were collected from Dong’an County in three field trips during April, July and October 2024. The habitat of the new species was investigated in the field. Descriptions are based on dried collections, except for flower colour.

### ﻿DNA extraction, amplification and sequencing

We collected fresh green leaves of this new species from Hunan, China. Total genomic DNA was extracted from silica-dried leaves with a plant genomic DNA kit (Tiangen Biotech Co., Ltd., Beijing, China). We used the nuclear ribosomal DNA internal transcribed spacer (ITS) for phylogenetic analyses. The universal primers ITS4 and ITS5 (White et al. 1990) were used to amplify the nuclear ribosomal ITS region. Amplification was undertaken using a volume of 25 µl with 20 µl Green Taq Mix (Novogene, China), 1.5 µl forward primer, 1.5 µl reverse primer, and 2 µl total DNA. The amplification of the ITS region was obtained by initial denaturation for 2 min at 98 °C, followed by 35 cycles of 10 s at 98 °C,15 s at 52 °C, and 15 s at 72 °C, and then a final extension of 5 min at 72 °C. All PCR products were separated using a 1.5% (w/v) agarose TAE gel and sent to Sangon (Nanjing, China) for sequencing.

### ﻿Phylogenetic analysis

To confirm the phylogenetic position of this species, 28 ITS belonging to 15 species with accession numbers were obtained from GenBank. A total of 29 taxa were sampled for phylogenetic analysis, including 27 taxa and 14 species from *Pternopetalum*. Two species *Oenanthehookeri* C. B. Clarke and *Siumsuave* Walter, served as outgroups.

We used SeqMan7 (Burland 2000) to assemble ITS sequences. The sequences were aligned using MAFFT v7.221 (Katoh and Standley 2013). The alignment was employed to reconstruct the phylogenetic tree using Maximum-Likelihood (ML) and Bayesian Inference (BI) methods. For ML analyses, the software RAxML v8.2.8 (Stamatakis 2014) was used to construct the phylogenetic trees with the GTR model and 1000 bootstrap (BS) replicates. Bayesian inference (BI) analyses were conducted by MrBayes version 3.2.7 (Ronquist et al. 2012) with the best-fit substitution model (GTR+G+I) determined by Modeltest v3.7 (Posada and Crandall 1998). Markov Chain Monte Carlo (MCMC) search was performed for 1 × 10^8^ generations, sampling every 1000 generations.

## ﻿Results and discussion

Molecular phylogenetic analyses based on the internal transcribed space (ITS) region showed that *P.shunhuangensis* is sister to *P.tanakae* (Franch. & Sav.) Hand.-Mazz. and *P.gracillimum* (H. Wolff) Hand.-Mazz. (Fig. 4). Thus, both morphological and phylogenetic evidence suggested that *P.shunhuangensis* is a distinct species of *Pternopetalum*.

After examining of herbarium specimens, we found that two other collections of the same species were collected in 1962 and 1984 respectively from Ziyun Mountain, which is part of the Shunhuang Mountain. However, they were misidentified as *P.heterophyllum* Hand.-Mazz. and *P.filicinum* (Franch.) Hand.-Mazz respectively. Both of the two species have already been treated as synonym of *P.tanakae* (Franch. & Sav.) Hand.-Mazz by Wang (2012). This treatment is widely accepted. The new species resembles *P.tanakae* in possessing the minute calyx teeth and filiform ribs, but differs from the latter by roots without tubercles at nodes, fewer basal leaves, cauline leaves and stems and elongate styles (Fig. 3F). In fact, the length of ultimate segments of the cauline leaves in the two species are distinctly different (3–5 mm in *P.shunhuangensis* vs.10–25 mm in *P.tanakae*; Fig. 3D).

As no population assessment has been conducted for this species in its entire range, it is best to assign a conservation status of ‘Data Deficient’ (DD) for this species (IUCN Standards and Petitions Committee 2022).

### ﻿Taxonomic treatment

#### ﻿ Pternopetalum shunhuangensis

Taxon classificationPlantaeApialesApiaceae

W.Zhou & L.Wu sp. nov.

99E01637-B24A-512E-8BAB-785B600BD349

urn:lsid:ipni.org:names:77358254-1

Figs 1–3

##### Type.

China • Hunan Province, Yongzhou City, Dong’an County, Hunan Dong’an Shunhuang Mountain National Nature Reserve, on rocks densely covered with moss in forests, at an altitude ca 1600 m a.s.l., 23 Apr. 2024, *Lei WU*, *Yan-Jie Yang*, *Zi-Lin Feng SHS0001* (holotype NAS); 19 July 2024, *Lei WU*, *D. Deng*, *Zi-Lin Feng SHS1749* (paratype CSFI).

**Figure 1.**
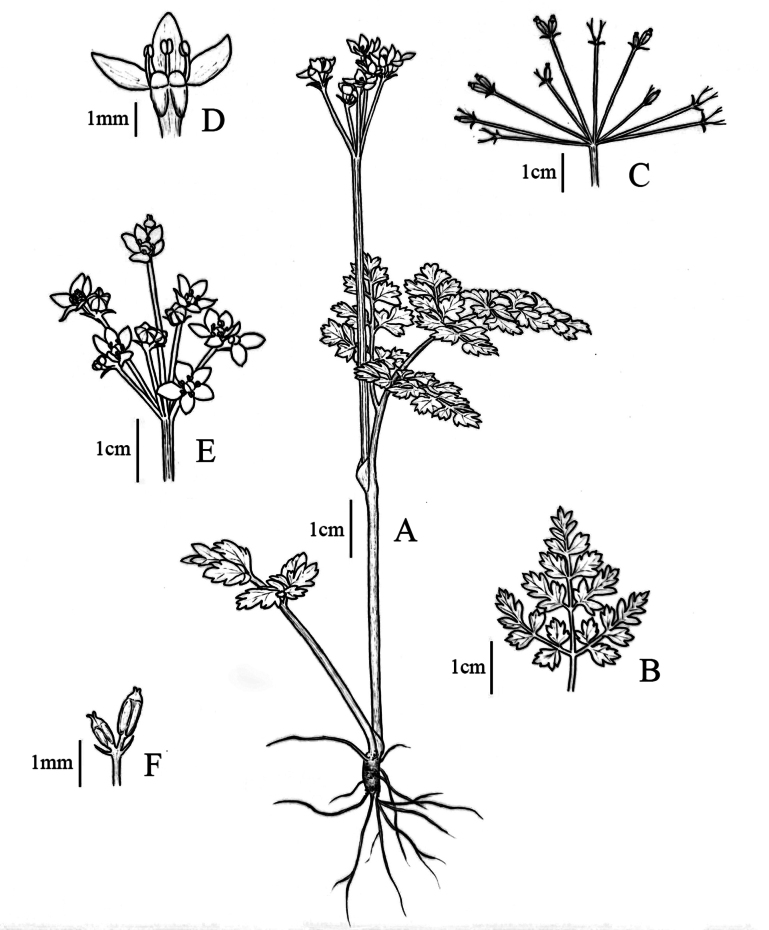
Illustration of *Pternopetalumshunhuangensis* W. Zhou & L. Wu **A** habit **B** another type of basal leaf **C** umbel in fruit **D** flower with two petals removed to show stamens and stylopodium **E** umbel in flower **F** mericarps. (Drawn by B. S. Li).

##### Etymology.

The species epithet is derived from the type locality, Shunhuang Mountain. Its Chinese name is given as 舜皇囊瓣芹 [Pinyin: shùn huáng náng bàn qín].

**Figure 2.**
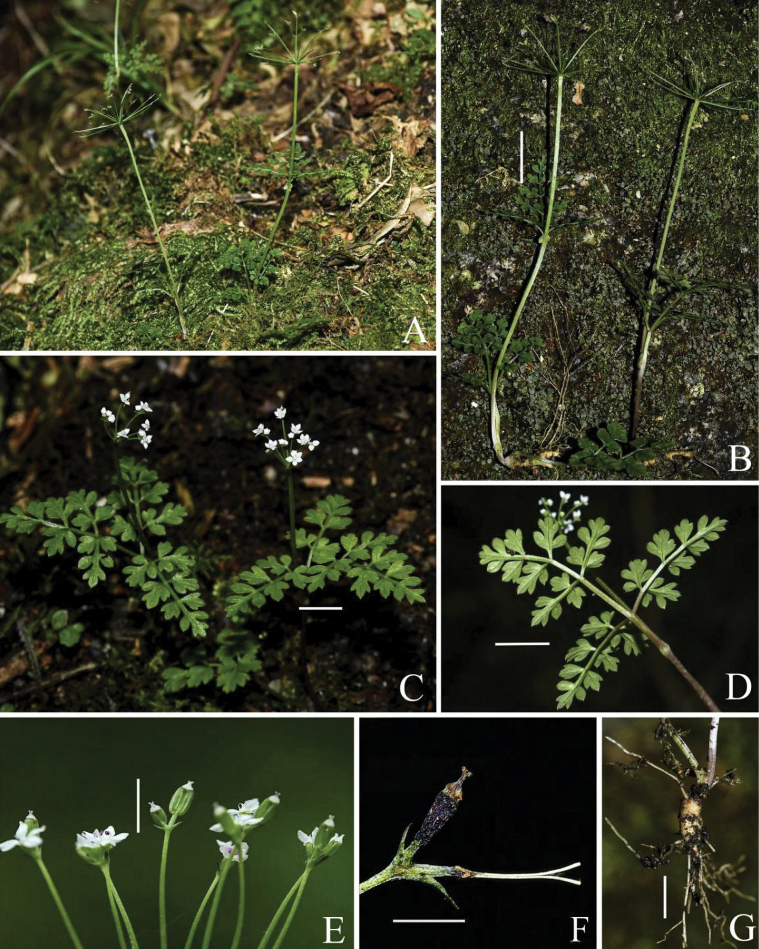
*Pternopetalumshunhuangensis* W. Zhou & L. Wu **A** microhabitat **B** habit **C** flowering plant **D** abaxial surface of cauline leaf **E** umbels in fruit **F** mature fruit **G** root. Scale bars: 2 cm (**B**); 1 cm (**C**–**D**, **G**); 2 mm (**E**–**F**).

##### Diagnosis.

*Pternopetalumshunhuangensis* differs from *P.tanakae* (Franch. & Sav.) Hand.-Mazz. by its roots without tubercles at nodes, fewer basal leaves, unelongated ultimate segments of cauline leaves, elongate styles and terminal umbels. A more detailed comparison between the two species is presented in Table 1.

**Table 1.** Comparison of *Pternopetalumshunhuangensis* and *P.tanakae*. Morphological data for *P.tanakae* are obtained from Pu and Phillippe (2005).

Characters	*P.shunhuangensis*	*P.tanakae*
Height (cm)	12–16	10–40
Root	without tubercles at nodes	with tubercles at nodes
Stem	1, unbranched	1–2, 1–2 branched or unbranched.
Basal leaves	0–2	2–4
Cauline leaves	1, ultimate segments flabelliform or diamond	1–3, ultimate segments lanceolate or elongate-linear
Position of umbels	terminal	lateral and terminal
Number of flowers in each umbellule	2	1–3
Style	longer than stylopodium	shorter than stylopodium

**Figure 3.**
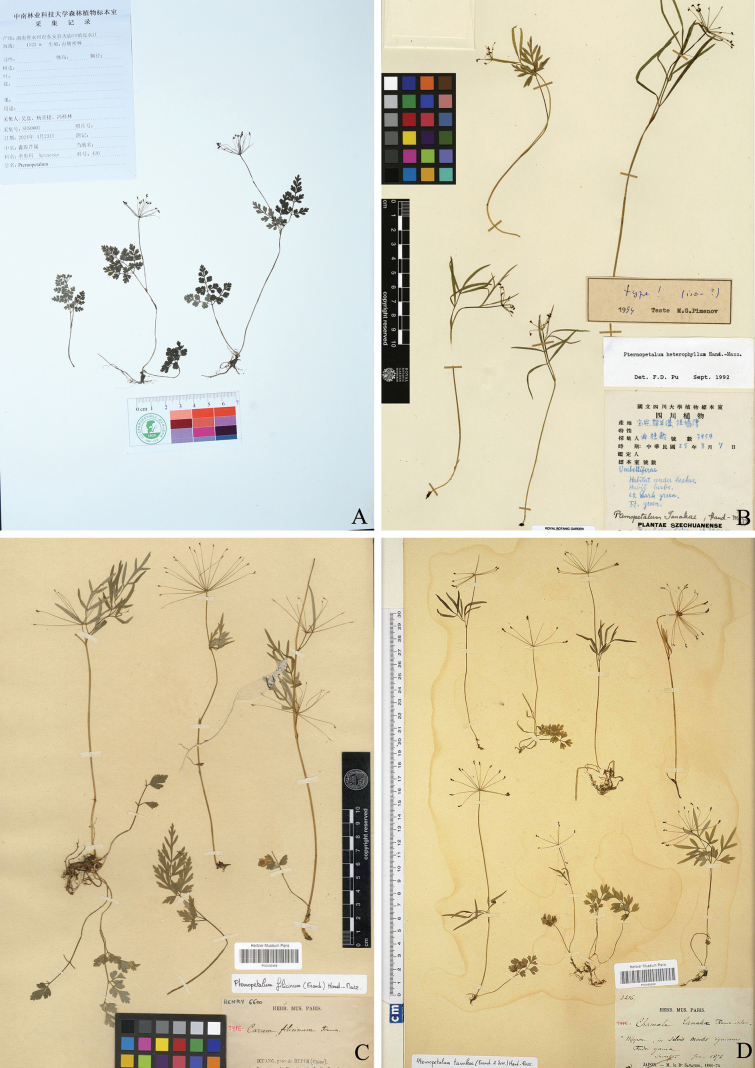
Type specimens of *Pternopetalumshunhuangensis*, *P.heterophyllum*, *P.filicinum* and *P.tanakae*. **A** holotype of *P.shunhuangensis* (NAS) **B** holotype of *P.heterophyllum* (E00265241) **C** isotype of *P.filicinum* (P00432458) **D** holotype of *P.tanakae* (P00495952).

Plants 12–16 cm high. Taproot fusiform. Stem 1, erect, slender, unbranched, glabrous. Basal leaves 0–1(2), petiolate; petioles 3–5 cm; blade ovate-triangular, 1.5–3 × 2.5–3 cm, ternate or ternate-2-pinnate; ultimate segments flabelliform or diamond, 3–8 × 2–7 mm. Cauline leaves 1, ternate-2-pinnate, petioles 0.5–4 cm; 2–4.5 × 3–6 cm, ultimate segments flabelliform or diamond, 2–5 × 2–6 mm. Umbels 1–3 cm across in flower, to 5 cm in fruit; bracts absent; rays 6–20, 1.5–2.5 cm; bracteoles 2–3; umbellules 2-flowered; pedicels 0.2–1 mm long. Calyx teeth minute, or obsolete. Petals white, broad ovate, 1.5–1.8 × 1.2–1.5 mm; stylopodium conic; styles elongate, erect, longer than the stylopodium; stylopodium plus style ca 0.8mm, styles ca. 0.5 mm. Fruit oblong ovoid, 2–2.5 × 1–1.5 mm; ribs filiform; vittae 1–2 in each furrow, 2 on commissure.

**Figure 4.**
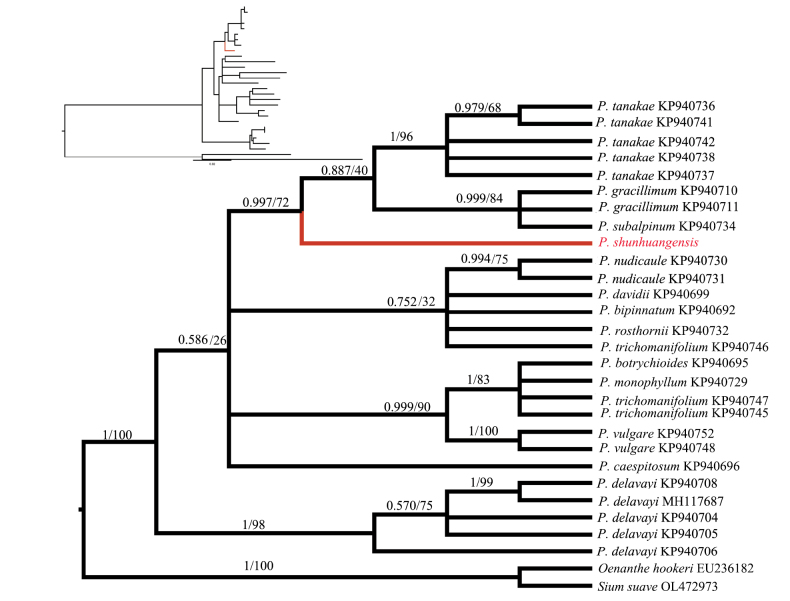
Phylogenetic tree inferred from the nrDNA ITS sequences. Bayesian posterior probability values (PP) / Bootstrap support values (BS) are shown on the branches. Only branches with PP > 0.5 are shown.

##### Phenology.

*Pternopetalumshunhuangensis* is flowering from April to May, and fruiting from June to July.

##### Additional specimens examined.

*Pternopetalumshunhuangensis* (paratypes): China • Hunan Province: Xinning County, 21 Oct. 1962 *Lin-Han Liu 15250* (NAS, WUK); Xinning County, Ziyun mountain, 9 Sep. 1984 *Ziyunshan Team 301*, *981* (PE).

*P.tanakae*: Japan • Silvis montis ignivomi, Fudsi Yama, Jun. 1874 *Savatier 3436* (holotype P, image).

*P.heterophyllum*: China • Hunan Province: Xinning County, Huping mountain, 10 Jul. 1987 *Hupingshan Team 1318* (PE); Sichuan Province: Baoxing County, 7 Agu. 1936 *Gui-ling Qu 3454* (holotype E, image).

*P.filicinum*: China • Hubei Province: Ichang City, *A. Henry 6600* (isotype P, image).

## Supplementary Material

XML Treatment for Pternopetalum shunhuangensis

## ﻿Additional information

### Author ORCIDs

Wei Zhou https://orcid.org/0000-0003-1627-1529

Zi-Lin Feng https://orcid.org/0009-0000-4283-5135

Long-Ping Tang https://orcid.org/0009-0003-6590-1491

Du Deng https://orcid.org/0009-0000-5476-4098

Bao-cheng Wu https://orcid.org/0009-0006-1719-3517

Lei Wu https://orcid.org/0000-0003-1451-7855

### Data availability

All of the data that support the findings of this study are available in the main text or Supplementary Information.

## Supplementary materials

Supplementary material 1 Supplementary data Data type fasta https://binary.pensoft.net/file/1278800This dataset is made available under the Open Database License (http://opendatacommons.org/licenses/odbl/1.0/). The Open Database License (ODbL) is a license agreement intended to allow users to freely share, modify, and use this Dataset while maintaining this same freedom for others, provided that the original source and author(s) are credited.Wei Zhou, Zi-Lin Feng, Long-Ping Tang, Du Deng, Bao-cheng Wu, Lei Wu
